# Do polymorphisms in protein kinase catalytic subunit alpha-1 gene associated with cancer susceptibility? a meta-analysis and systematic review

**DOI:** 10.1186/s12881-018-0704-8

**Published:** 2018-10-19

**Authors:** Jialin Meng, Xinyao Fan, Meng Zhang, Zongyao Hao, Chaozhao Liang

**Affiliations:** 1 Department of Urology, The First Affiliated Hospital of Anhui Medical University, No. 218th, Jixi Road, Hefei, 230022 Anhui China; 2 Institute of Urology, Anhui Medical University, No. 218th, Jixi Road, Hefei, 230022 Anhui China; 3 Anhui Province Key Laboratory of Genitourinary Diseases, Anhui Medical University, No. 218th, Jixi Road, Hefei, 230022 Anhui China; 4 Graduate School of Anhui Medical University, No. 81th, Meishan Road, Hefei, 230032 Anhui China

**Keywords:** *PRKAA1*, Polymorphism, Cancer, Meta-analysis

## Abstract

**Background:**

Currently, several studies have demonstrated that *PRKAA1* polymorphisms conduce to the development of cancer. *PRKAA1* gene encodes the AMP-activated protein kinase summit-α1, and plays an important role in cell metabolism. Thus, we performed a systematic review and meta-analysis of all enrolled eligible case-control studies to obtain a precise correlation between *PRKAA1* polymorphism and cancer susceptibility.

**Methods:**

Extensive retrieve was performed in Web of Science, Google Scholar, PubMed, EMbase, CNKI and Wanfang databases up to August 26, 2018. Odds ratios (ORs) and 95% CIs were performed to evaluate the overall strength of the associations in five models, as well as in subgroup analyses, stratified by ethnicity, cancer type or source of control. Q-test, Egger’s test and Begg’s funnel plot were applied to evaluate the heterogeneity and publication bias. In-silico analysis was performed to demonstrate the relationship of *PRKAA1* expression correlated with cancer tissues and survival time.

**Results:**

Twenty-two case-control studies from 14 publications were enrolled, with 17,068 cases and 20,871 controls for rs13361707, and 2514 cases and 3193 controls for rs10074991. Overall, we identified that the *PRKAA1* rs13361707 polymorphism is not significantly associated with cancer susceptibility under all five genetic models. For rs10074991, we revealed a significant decrease risk in allelic comparison model (B vs. A: OR = 0.774, 95% CI = 0.642–0.931, *P*_*Adjust*_ = 3.376*10^− 2^), heterozygote comparison model (BA vs. AA: OR = 0.779 95%CI = 0.691–0.877, *P*_*Adjust*_ = 9.86*10^− 10^;), and dominant genetic model (BB + BA vs. AA: OR = 0.697 95%CI = 0.533–0.912, *P*_*Adjust*_ = 4.211*10^− 2^;). Evidence from TCGA database and GTEx projects indicated that the expression of *PRKAA1* in gastric cancer tissue is higher, compared to normal stomach tissue, as well as it in breast cancer and esophageal squamous cell carcinoma. However, the Kaplan-Meier estimate showed that there is no significant difference of OS and RFS between the low and high *PRKAA1* TPM groups in gastric cancer, breast cancer, and esophageal carcinoma.

**Conclusions:**

To sum up, *PRKAA1* rs13361707 polymorphism is not participant with the increased risk of cancer, while the A allele of *PRKAA1* rs10074991 revealed a significant decrease risk.

**Electronic supplementary material:**

The online version of this article (10.1186/s12881-018-0704-8) contains supplementary material, which is available to authorized users.

## Background

It is well known that hereditary materials and environmental aspects could influence the risks and take a critical part in the tumorigenesis of numerous cancers [1, 2], nevertheless, the risk attributable to each cancer is indistinct. Of them, an important one is AMP-activated protein kinase (AMPK). As a heterotrimeric protein complex, AMPK always made up of one α, β and γ subunit, respectively, and the 39 kb-long encodes gene (*PRKAA1)* of α-subunit is located at chromosome 5p12.1 [3, 4]. Numerous pivotal cell metabolic enzymes are regulated by AMPK, through its function of Ser/Thr phosphorylation [5].AMPK induce the important inhibition of tumorigenesis through multifaceted ways. Firstly, AMPK activation contributes to the inhibition in fatty acid biosynthesis and cholesterol biosynthesis, as well as to the promotion of fatty acid oxidation, therefore, against to the intracellular lipid accumulation and insulin resistance development in non-adipose tissues [6]. Secondly, another important tumorigenesis related signaling pathway, the mammalian target of rapamycin complex 1 (mTORC1), can be inhibited by the activation of AMPK [7]. Thirdly, AMPK activation can result in the G1 phase cell cycle arrest, and further impact the cell proliferation through the p53-p21 axis [8].

Most of the publications concerned about *PRKAA1* polymorphisms focused on its significantly positive association with gastric cancer (GC) [9–11], as well as on breast cancer [12], but there are also some negative results [13–16]. These conflicting results might be partially affect by the cancer type, origin of control, or sample size. Herein, we managed the meta-analysis to assess whether *PRKAA1* polymorphisms affect susceptibility of cancer.

## Methods

### Identification and selection of eligible studies

Comprehensive literature search on Web of Science, Google Scholar, PubMed, EMbase, CNKI and Wanfang databases was conducted to draw out all eligible case-control studies, and the latest search date is August 26, 2018. The following are valid keyword search strings: (PRKAA1) AND (polymorphism OR SNP OR variant OR mutation OR allele) AND (cancer OR tumor OR tumour OR carcinoma OR neoplasm OR malignancy). What’s more, we also manually retrieved the references of reviews or original research on this issue to identify additional studies. For these republished and overlapping studies, we enrolled the most recently published articles or case-control studies with a maximum number of subjects.

### Enrolled criteria and excluded criteria

All the eligible studies were enrolled following the details: 1) assess the correlation between the polymorphisms in *PRKAA1* and cancer susceptibility; 2) case-control studies; 3) demonstrate the frequency of genotypes of all cases and controls, or could obtain it via calculating. However, studies would be excluded if they meet the items: 1) animal studies, meta-analysis, comments, reviews or case reports; 2) no efficient data of the genotype frequency; 3) repetitive publications; 4) the research contents were concerned about other disorders instead of cancers.

### Extraction of data

Extraction of data were independently completed by two authors (Jialin Meng, Xinyao Fan), and all disagreements were finally obtained a consensus after discussion. The following items from the eligible case-control studies were extracted: SNP code, first author, year of publication, ethnicity, cancer type, genotyping method, source of control and genotype frequency. For the control group of each study, we definition it as population-based or hospital-based from whether it is collected from physical examination or just no-cancer patients, and ethnicity was distinguished as “Caucasian” or “Asian”.

### Statistical analysis

We performed the meta-analyses in the pool, ORs with corresponding 95% CI was recorded to evaluated the strength of the correlation between *PRKAA1* polymorphisms (rs13361707, rs10074991) and cancer susceptibility, the *P* value was adjusted by Bonferroni corrections, *P*_*Adjust*_ = *P*_*Z*_ * 5 models [17]. We pooled the ORs for allelic comparison model (B vs. A), heterozygote comparison model (BA vs. AA), homozygote comparison model (BB vs. AA), dominant genetic model (BB + BA vs. AA) and recessive genetic model (BB vs. BA +AA). We further applied Q-test to assess the between study heterogeneity [18]. When the *P* value of the Q-test was > 0.1, we selected the Mantel-Haenszel method (fixed model) to evaluate the pooled OR estimate; On the contrast, the Der Simonian and Laird method (random-effect model) was preferred to evaluated the *P* value < 0.1 group [19, 20]. In the subgroup of ethnicity and source of control, stratified analyses were also conducted. The Hardy-Weinberg equilibrium (HWE) for the control group was assessed by a professional web-based program (https://wpcalc.com/en/equilibrium-hardy-weinberg/), the *P*-value > 0.05 suggested a HWE balance for the control group. In order to appraise the stability of the meta-analyses, we managed sensitivity analyses by excluding each case-control study and observe whether it influence the pooled ORs and 95% CI [21]. What’s more, Begg’s funnel plot and Egger’s test was also conducted to avoid any potential publication bias [22, 23]. All the analysis results were calculated by STATA 12.0 (version 12.0; Stata Corporation). and *P*<0.05 was regarded as statistically significant.

### In-silico analysis

To further explore whether the expression of PRKAA1 affect tumorigenesis, we tried to search some evidence from the public TCGA database and GTEx projects, and a newly developed interactive website, GEPIA (http://gepia.cancer-pku.cn/), was applied to draw out it [24].

## Results

### Characteristics of studies

**Fig. 1:**
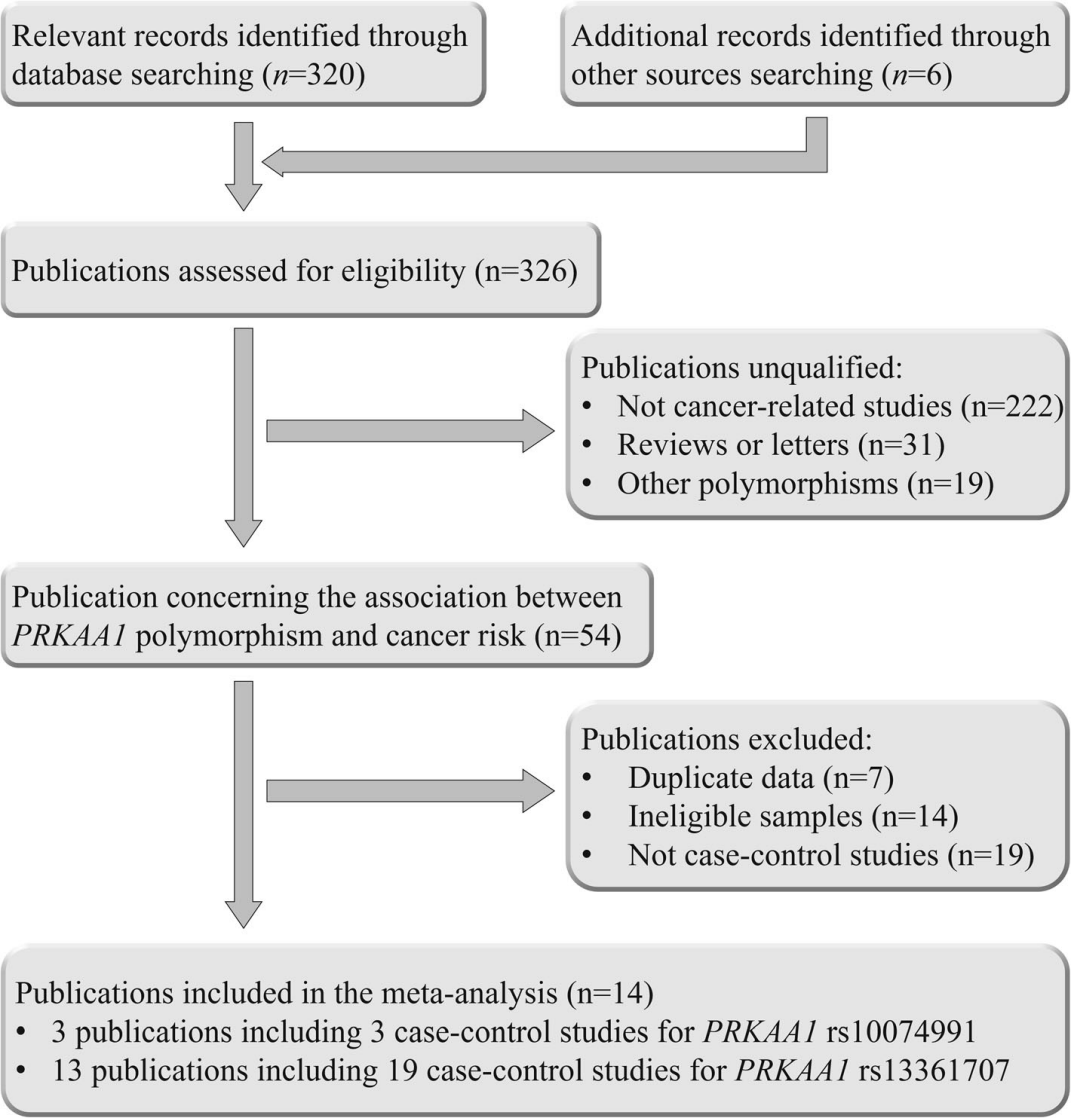
Flow chart showing the study selection procedure

Figure 1 displays the process of searching, a total of 320 publications was firstly retrieved from PubMed, EMbase, Web of Science, CNKI and Wanfang databases. After reviewed the abstract, 54 publications were selected for a further evaluation, however, 30 of them were expurgated because of that they were duplicated studies, case-only studies, or less of efficient data. Finally, 22 case-control studies from 14 publications were enrolled in our study, 17,068 cases and 20,871 controls for rs13361707 [9–16, 25–30], while 2514 cases and 3193 controls for rs10074991 [10, 12, 27] (Table 1**,** Additional file 1: Table S1). There are 20 case-control studies from Asian descendants, and 2 from Caucasian. Diagnose of tumor was confirmed histologically or pathologically. As to HWE, only three studies were not conformed to it, while the other 19 case-control studies conformed to it. In addition, the result of appraise the quality of study by Newcastle-Ottawa Scale (NOS) is filled in Additional file 1: Table S2 [31], and Additional file 1: Table S3 shows the result of PRISMA2009 checklist [32].

**Table 1:** Characteristics of the enrolled studies on PRKAA1 polymorphisms and cancer

SNP	First author	Year	Ethnicity	Genotyping Method	Source of Control	Cancer Type	Csae			Control			HWE
							PAA	PAB	PBB	HAA	HAB	HBB	
rs10074991	Campa et al.	2011	Caucasian	HapMap	HB	BC	654	445	94	960	764	149	Y
rs10074991	Kim et al.	2014	Asian	GoldenGate assay	PB	GC	136	242	97	94	244	136	Y
rs10074991	Eom et al.	2016	Asian	GoldenGate assay	PB	GC	248	421	177	169	429	248	Y
rs13361707	Shi et al.	2011	Asian	AGWHSA 6.0 chips	PB	GC	160	517	302	607	1154	507	Y
rs13361707	Shi et al.	2011	Asian	AGWHSA 6.0 chips	PB	GC	371	941	561	578	1034	464	Y
rs13361707	Shi et al.	2011	Asian	AGWHSA 6.0 chips	PB	GC	237	675	480	392	745	376	Y
rs13361707	Shi et al.	2011	Asian	AGWHSA 6.0 chips	PB	GC	223	447	225	713	1616	898	Y
rs13361707	Shi et al.	2011	Asian	AGWHSA 6.0 chips	PB	GC	724	1221	459	713	1616	898	Y
rs13361707	Li et al.	2013	Asian	TaqMan	PB	GC	97	167	71	67	165	102	Y
rs13361707	Song et al.	2013	Asian	HRM-PCR	PB	GC	909	1654	682	377	846	477	Y
rs13361707	Dai et al.	2014	Asian	TaqMan	PB	ESCC	460	1054	558	507	1144	603	Y
rs13361707	Wu et al.	2014	Asian	Multiplex SNaPshot SNP	PB	GC	54	115	48	86	209	133	Y
rs13361707	Kim et al.	2014	Asian	GoldenGate assay	PB	GC	137	241	97	96	242	135	Y
rs13361707	Sun et al.	2014	Caucasian	TapMan	HB	GC	79	45	6	68	48	8	Y
rs13361707	Dong et al.	2015	Asian	iMLDR	PB	GC	37	68	62	54	91	41	Y
rs13361707	Dong et al.	2015	Asian	iMLDR	PB	NSCLC	41	71	46	54	91	41	Y
rs13361707	Dong et al.	2015	Asian	iMLDR	PB	ESCC	33	51	23	54	91	41	Y
rs13361707	Qiu et al.	2015	Asian	TaqMan	PB	GC	344	571	209	273	565	356	Y
rs13361707	Zhang et al.	2016	Asian	MALDITOF	HB	GC	23	27	10	10	34	16	Y
rs13361707	Eom et al.	2016	Asian	GoldenGate assay	PB	GC	249	421	176	174	424	248	Y
rs13361707	Yuan et al.	2016	Asian	PCR	HB	GC	31	59	26	28	49	25	Y
rs13361707	Cai et al.	2017	Asian	KASP	PB	GC	172	213	88	98	246	143	Y

*GC* Gastric cancer, *BC* Breast cancer, *ESCC* Esophageal squamous cell carcinoma, *NSCLC* Non-small cell lung cancer, *HB* Hospital based, *PB* Population based, *HWE* Hardy Weinberg Equilibrium

### Main results

**Fig. 2:**
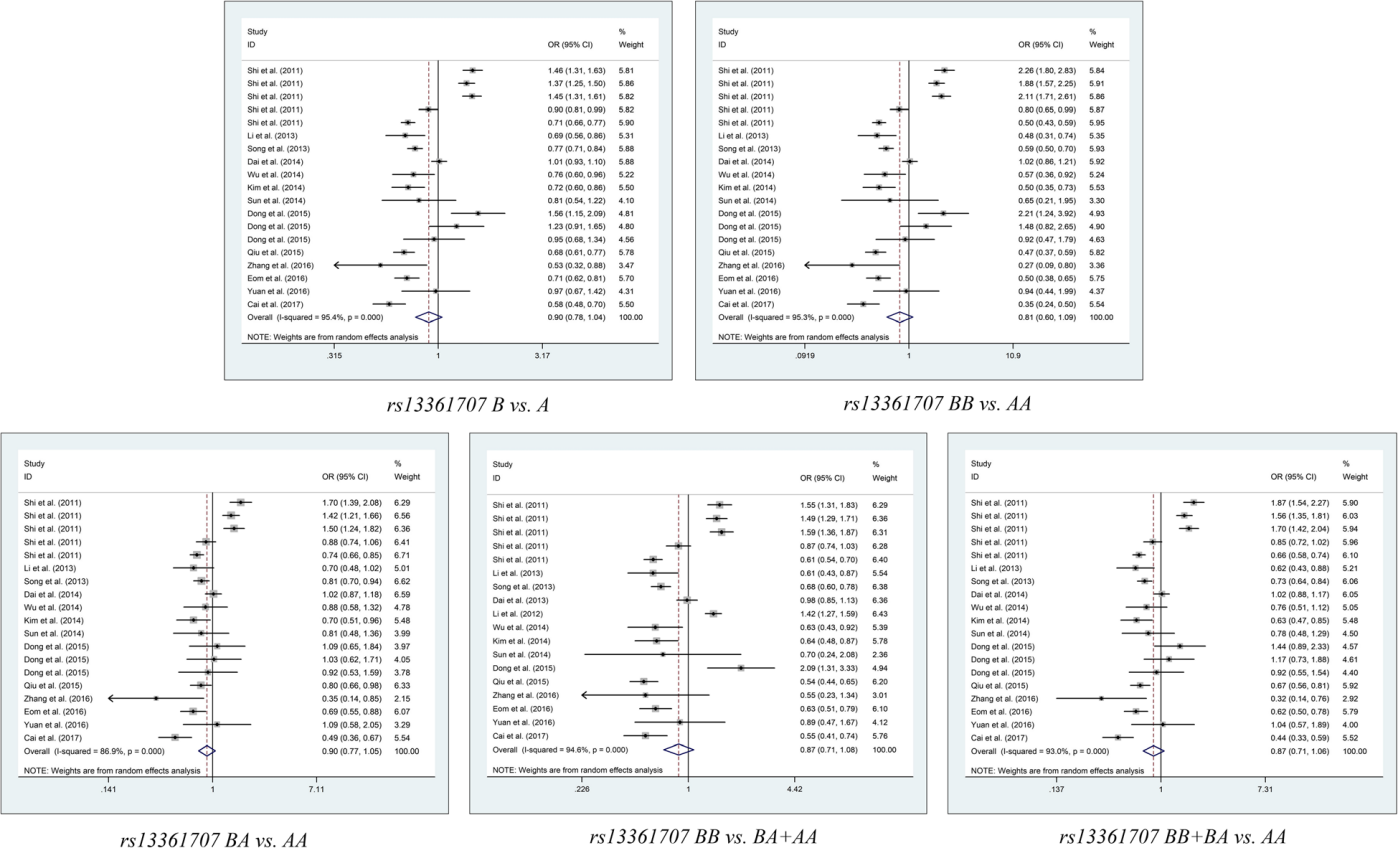
Meta-analysis of the association between *PRKAA1* rs10074991 polymorphism and cancer risk

**Fig. 3:**
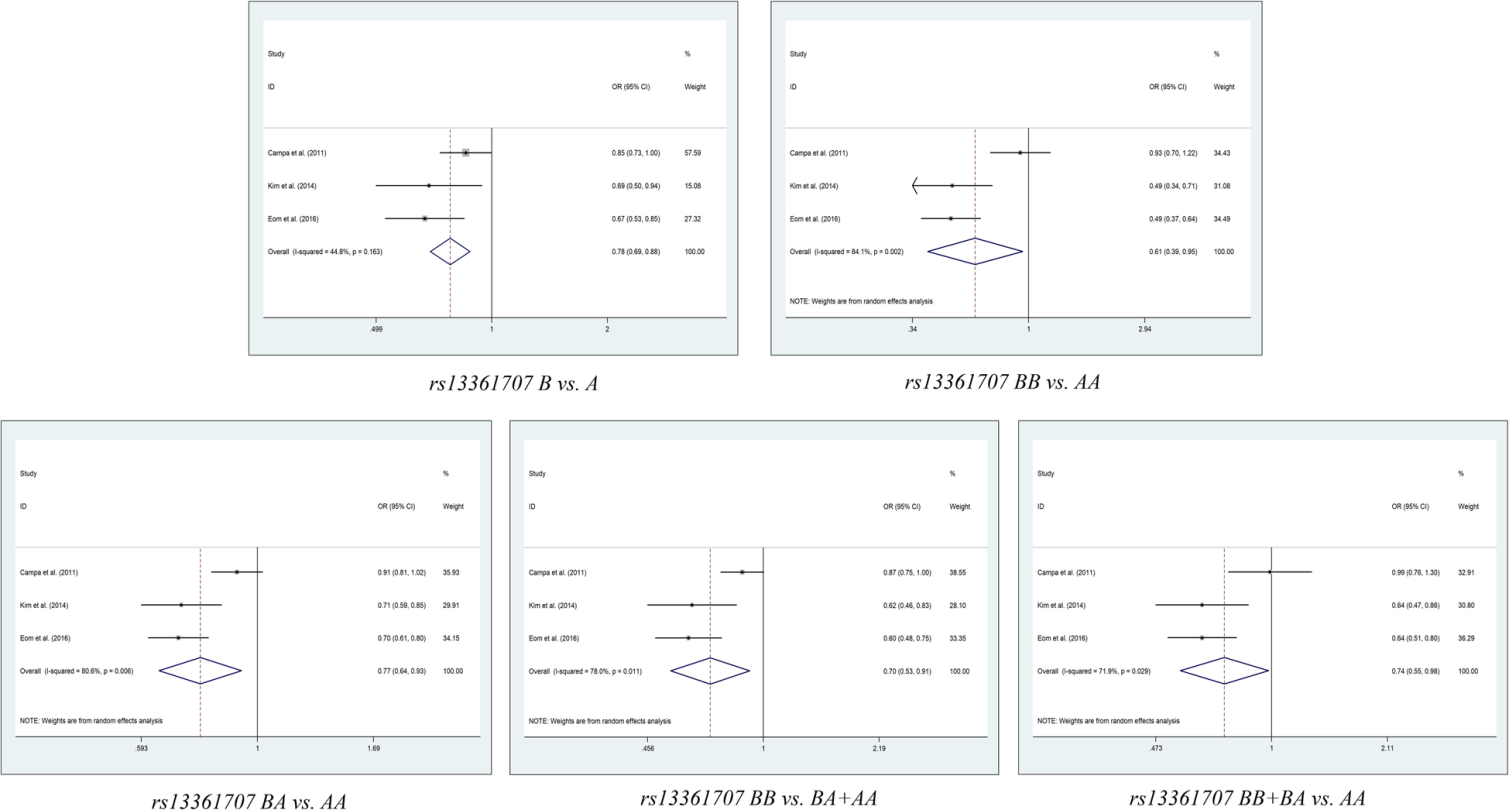
Meta-analysis of the association between *PRKAA1* rs13361707 polymorphism and cancer risk

Table 2 shows the summarized details and results of the meta-analyses. We identified that the rs13361707 polymorphism is not related to the susceptibility of cancer under all five genetic models in the overall population (B vs. A: OR = 0.900, 95%CI = 0.776–1.042, *P*_*Adjust*_ = 0.795; BB vs. AA: OR = 0.810, 95% CI = 0.601–1.092, *P*_*Adjust*_ = 0.830; BA vs. AA: OR = 0.900, 95% CI = 0.768–1.054, *P*_*Adjust*_ = 0.960; BA+AA vs. AA: OR = 0.569, 95% CI = 0.711–1.061, *P*_*Adjust*_ = 0.840; and BB vs. BA+AA: OR = 0.965, 95% CI = 0.714–1.071, *P*_*Adjust*_ = 0.965), and the stratified analysis also indicated no relationships in the subgroups of cancer type, ethnicity and source of control (Fig. 2). For rs10074991, we revealed a significant decrease risk in allelic comparison model (B vs. A: OR = 0.774, 95% CI = 0.642–0.931, *P*_*Adjust*_ = 3.376*10^− 2^), heterozygote comparison model (BA vs. AA: OR = 0.779 95%CI = 0.691–0.877, *P*_*Adjust*_ *=* 1.948*10^− 4^;), and dominant genetic model (BB + BA vs. AA: OR = 0.697 95%CI = 0.533–0.912, *P*_*Adjust*_ = 4.211*10^− 2^;) (Fig. 3), and the subgroup of Asian people in rs10074991 shown a prevent potential for tumorigenesis in all five genetic models (B vs. A: OR = 0.704, 95%CI = 0.632–0.785, *P*_*Adjust*_ = 1.085*10–9; BB vs. AA: OR = 0.489, 95% CI = 0.489 0.392–0.609, *P*_*Adjust*_ = 9.86*10–10; BA vs. AA: OR = 0.675, 95% CI = 0.558–0.816, *P*_*Adjust*_ = 2.473*10–4; BA+AA vs. AA: OR = 0.607, 95% CI = 0.507–0.727, *P*_*Adjust*_ = 2.691*10–7; and BB vs. BA+AA: OR = 0.638, 95% CI = 0.534–0.762, *P*_*Adjust*_ = 3.876*10–6).

**Table 2:** Results of meta-analysis for polymorphisms in and cancer susceptibility

Comparison	Subgroup	N	*P*_*H*_	*P*_*Z*_	*P*_*Adjust*_	Random (OR, 95%CI)	Fixed (OR, 95%CI)
rs10074991							
B VS. A	overall	3	0.006	6.752*10^−3^	3.376*10^–2*^	0.774 (0.642–0.931)	0.795 (0.735–0.861)
B VS. A	Asian	2	0.908	2.169*10^−10^	1.085*10^–9*^	0.704 (0.632–0.785)	0.704 (0.632–0.785)
BB vs. AA	overall	3	0.002	2.787*10^−2^	0.139	0.610 (0.393–0.946)	0.627 (0.528–0.745)
BB vs. AA	Asian	2	0.954	1.972*10^−10^	9.86*10^–10*^	0.489 (0.392–0.609)	0.489 (0.392–0.609)
BA vs. AA	overall	3	0.163	3.895*10^−5^	1.948*10^–4*^	0.755 (0.633–0.900)	0.779 (0.691–0.877)
BA vs. AA	Asian	2	0.903	4.946*10^−5^	2.473*10^–4*^	0.675 (0.558–0.816)	0.675 (0.558–0.816)
BB + BA vs. AA	overall	3	0.011	8.421*10^−3^	4.211*10^–2*^	0.697 (0.533–0.912)	0.752 (0.672–0.842)
BB + BA vs. AA	Asian	2	0.900	5.381*10^−8^	2.691*10^–7*^	0.607 (0.507–0.727)	0.607 (0.507–0.727)
BB vs. BA+AA	overall	3	0.029	3.610*10^−2^	0.181	0.737 (0.554–0.980)	0.729 (0.628–0.846)
BB vs. BA+AA	Asian	2	0.999	7.752*10^−7^	3.876*10^–6*^	0.638 (0.534–0.762)	0.638 (0.534–0.762)
rs13361707							
B VS. A	overall	19	6.416*10^−72^	0.159	0.795	0.900 (0.776–1.042)	0.931 (0.904–0.959)
B VS. A	Asian	18	1.644*10^−72^	0.188	0.940	0.904 (0.777–1.051)	0.932 (0.905–0.960)
B VS. A	GC	16	2.061*10^−72^	0.121	0.605	0.873 (0.736–1.037)	0.917 (0.889–0.947)
B VS. A	ESCC	2	0.749	0.884	1.000	1.006 (0.927–1.092)	1.006 (0.927–1.092)
B VS. A	PB	16	6.359*10^−73^	0.299	1.000	0.920 (0.786–1.077)	0.933 (0.906–0.961)
B VS. A	HB	3	0.167	0.061	0.305	0.773 (0.555–1.076)	0.793 (0.622–1.010)
BB vs. AA	overall	19	6.471*10^−71^	0.166	0.830	0.810 (0.601–1.092)	0.868 (0.819–0.921)
BB vs. AA	Asian	18	1.516*10^−71^	0.191	0.955	0.816 (0.602–1.106)	0.869 (0.819–0.922)
BB vs. AA	GC	16	2.211*10^− 71^	0.127	0.635	0.763 (0.539–1.080)	1.013 (0.859–1.196)
BB vs. AA	ESCC	2	0.765	0.875	1.000	0.844 (0.792–0.899)	1.013 (0.859–1.196)
BB vs. AA	PB	16	4.978*10^−72^	0.300	1.000	0.845 (0.615–1.161)	0.872 (0.822–0.925)
BB vs. AA	HB	3	0.182	0.088	0.440	0.592 (0.286–1.224)	0.630 (0.371–1.071)
BA vs. AA	overall	19	2.232*10^−20^	0.192	0.960	0.900 (0.768–1.054)	0.946 (0.899–0.996)
BA vs. AA	Asian	18	8.982*10^− 21^	0.224	1.000	0.904 (0.768–1.064)	0.948 (0.900–0.998)
BA vs. AA	GC	16	1.397*10^−21^	0.184	0.920	0.883 (0.734–1.061)	0.937 (0.886–0.990)
BA vs. AA	ESCC	2	0.727	0.912	1.000	1.008 (0.871–1.167)	1.008 (0.871–1.167)
BA vs. AA	PB	16	9.406*10^−21^	0.320	1.000	0.919 (0.778–1.086)	0.950 (0.902–1.001)
BA vs. AA	HB	3	0.121	0.155	0.775	0.730 (0.417–1.277)	0.768 (0.534–1.105)
BB + BA vs. AA	overall	19	1.835*10^−44^	0.168	0.840	0.869 (0.711–1.061)	0.916 (0.873–0.962)
BB + BA vs. AA	Asian	18	5.451*10^−45^	0.195	0.975	0.873 (0.71–1.072)	0.918 (0.874–0.963)
BB + BA vs. AA	GC	16	1.594*10^−45^	0.145	0.725	0.841 (0.666–1.062)	0.901 (0.856–0.949)
BB + BA vs. AA	ESCC	2	0.707	0.892	1.000	1.010 (0.879–1.159)	1.010 (0.879–1.159)
BB + BA vs. AA	PB	16	5.135*10^−45^	0.309	1.000	0.895 (0.723–1.108)	0.920 (0.876–0.966)
BB + BA vs. AA	HB	3	0.085	0.208	1.000	0.692 (0.389–1.228)	0.737 (0.522–1.041)
BB vs. BA+AA	overall	19	2.761*10^−46^	0.193	0.965	0.874 (0.714–1.071)	0.902 (0.860–0.946)
BB vs. BA+AA	Asian	18	7.547*10^−47^	0.217	1.000	0.878 (0.715–1.079)	0.902 (0.860–0.947)
BB vs. BA+AA	GC	16	1.034*10^−46^	0.135	0.675	0.836 (0.660–1.058)	0.882 (0.838–0.929)
BB vs. BA+AA	ESCC	2	0.892	0.917	1.000	1.007 (0.883–1.148)	1.007 (0.883–1.148)
BB vs. BA+AA	PB	16	7.502*10^−48^	0.293	1.000	0.891 (0.719–1.105)	0.904 (0.861–0.948)
BB vs. BA+AA	HB	3	0.681	0.219	1.000	0.747 (0.470–1.188)	0.746 (0.470–1.184)

*P*_*H*_ *P* value of Q test for heterogeneity test, *P*_*Z*_ means statistically significant, *P*_*Adjust*_ Multiple testing *P* value according to Bonferroni Correction, *H-B* Hospital based, *P-B* Population based, *HWE* Hardy Weinberg Equilibrium; Note: Heterogeneity was considered to be significant when the *P*-value was less than 0.1. If there was no significant heterogeneity, a fixed effect model (Der-Simonian Laird) was used to evaluate the point estimates and 95% CI; otherwise, a random effects model (Der-Simonian Laird) was used. And the *Pz* was calculated based on the actual model adopted. "*" indicated that *P*_*Adjust*_ value less than 0.05, and is considered as statistically significant

### Sensitivity and publication bias analysis

**Fig. 4:**
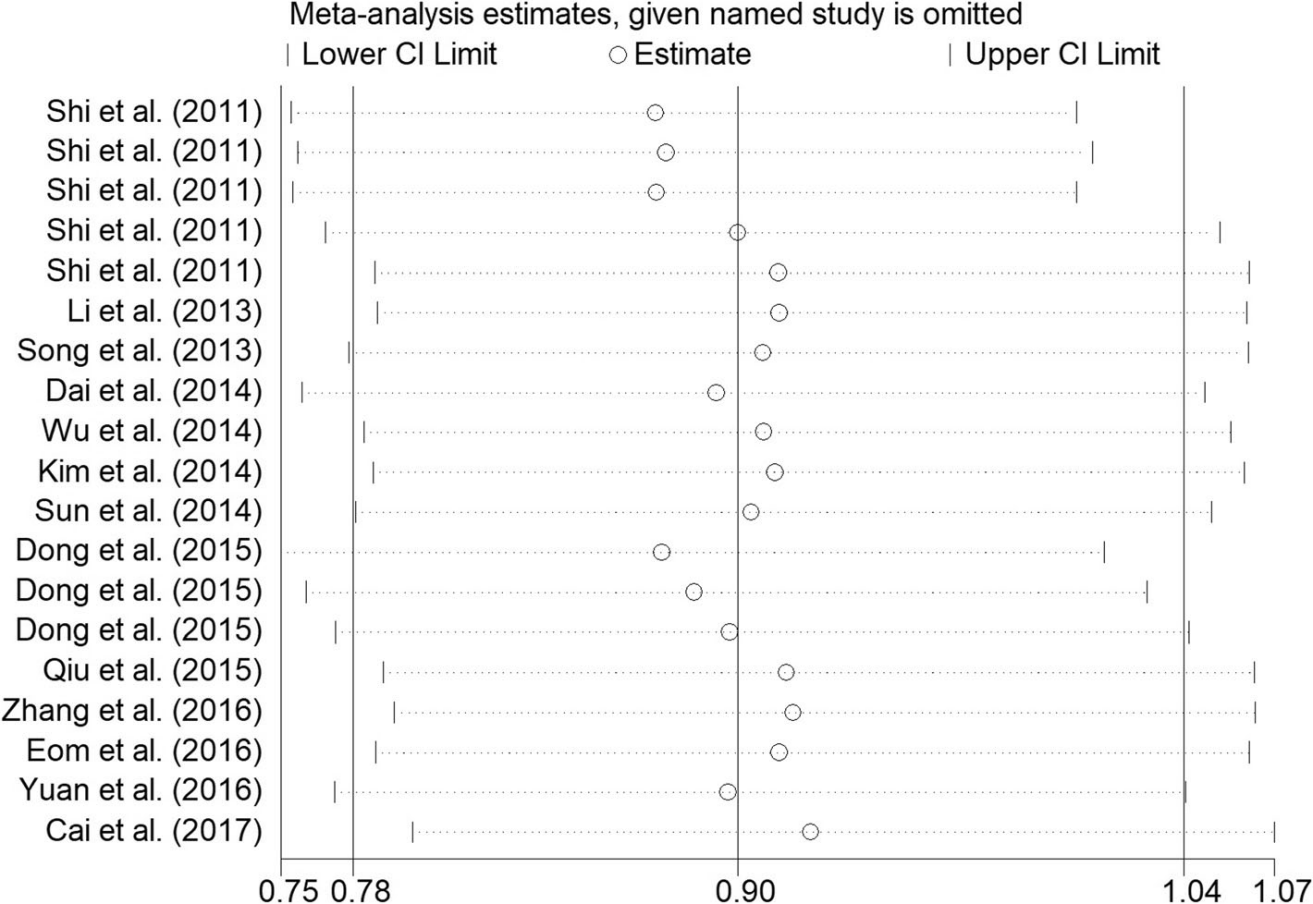
Sensitivity analysis of Overall OR Co-efficient for *PRKAA1* rs13361707 polymorphism (C vs. T). Results were calculated by omitting each study in turn. The two ends of the dotted lines represent the 95%CI

Sensitivity analysis by delete each signal study were performed to assess whether it influence the overall ORs results. The sensitivity analysis results conducted by all five genetic models were displayed in Fig. 4 and Additional file 1: Table S4, no signal study significantly influence the overall ORs results. On the besides, potential publication bias of enrolled case-control studies was appraised by Begg’s funnel plot and Egger’s test, no publication bias was revealed in both rs13361707 and rs10074991 (Additional file 1: Figures S1 and S2, Table S5).

To further explore whether the expression of PRKAA1 affect tumorigenesis, we tried to search some evidence from the public TCGA database and GTEx projects, and a newly developed interactive website, GEPIA (http://gepia.cancer-pku.cn/), was applied to draw out it.

### In-silico analysis

**Fig. 5:**
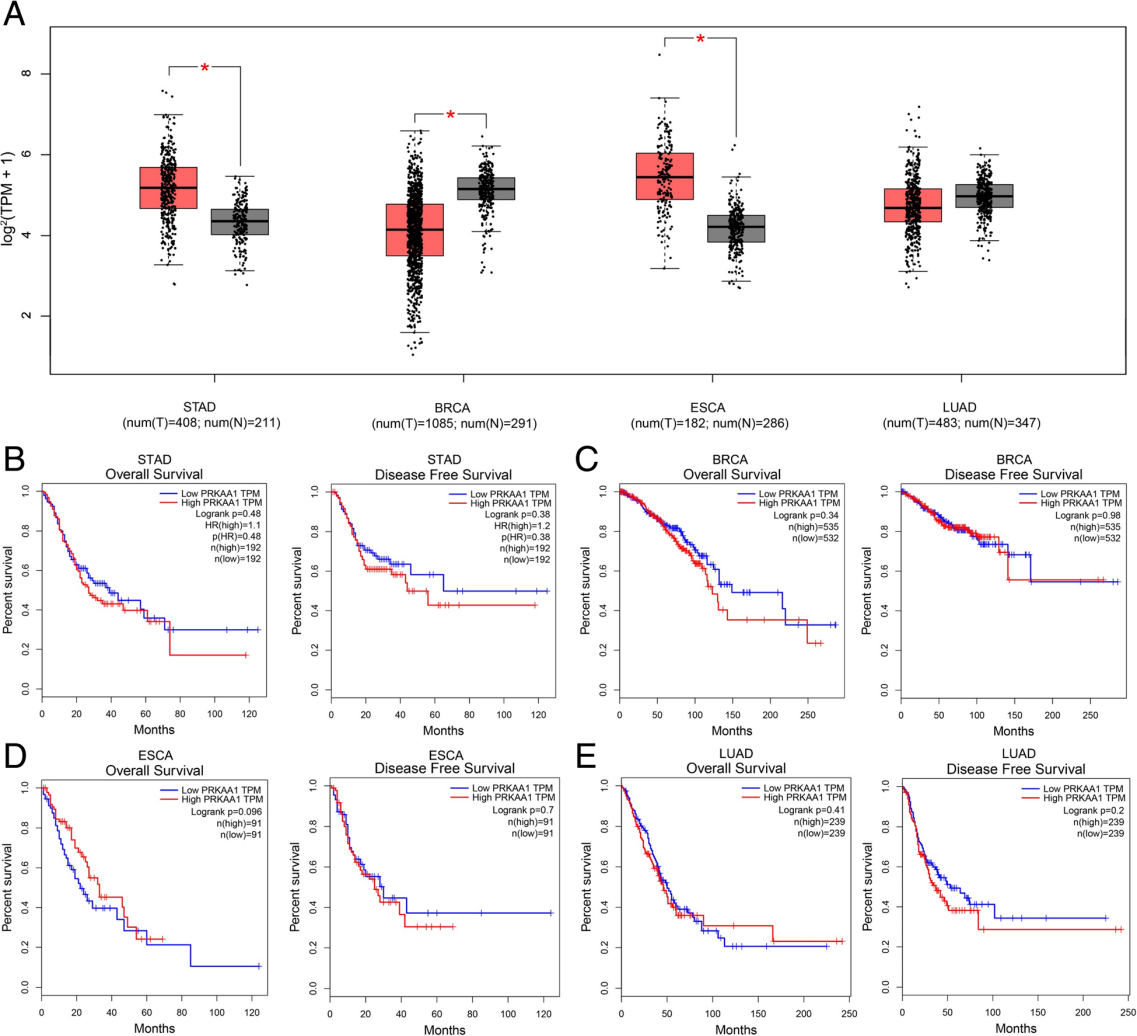
In-silico analysis of *PRKAA1* expression (**a**) The comparison of *PRKAA1* expression between tumor site and matched normal tissue from TCGA database. **b** The correlation between *PRKAA1* expression and overall survival time, disease free survival time in stomach adenocarcinoma (STAD). **c** The correlation between *PRKAA1* expression and overall survival time, disease free survival time in breast cancer (BRCA). **d** The correlation between *PRKAA1* expression and overall survival time, disease free survival time in esophageal carcinoma (ESCA). **e** The correlation between *PRKAA1* expression and overall survival time, disease free survival time in lung adenocarcinoma (LUAD)

Evidence from TCGA database and GTEx projects indicated that the expression level of *PRKAA1* in gastric cancer tissue is higher, when compared to normal stomach tissue (TPM = 54.3 vs. 29.2, *P* < 0.01,), as well as in breast cancer (TPM = 23.1 vs. 29.8, *P* < 0.01) and esophageal squamous cell carcinoma (TPM = 49.6 vs. 14.9, *P* < 0.01) (Fig. 5a). In addition, we analyzed whether the expression of *PRKAA1* affects the overall survival (OS) and disease free survival (RFS) of these four cancers. The Kaplan-Meier estimate showed that there is no significant difference of OS and RFS between the low and high *PRKAA1* TPM groups in gastric cancer (OS: Log-rank *P =* 0.48, RFS: Log-rank *P =* 0.38), breast cancer (OS: Log-rank *P =* 0.34, RFS: Log-rank *P =* 0.98), esophageal carcinoma (OS: Log-rank *P =* 0.096, RFS: Log-rank *P =* 0.7), and lung adenocarcinoma (OS: Log-rank *P =* 0.41, RFS: Log-rank *P =* 0.20).

## Discussion

AMPK is a highly conserved heterotrimeric Ser/Thr kinase, it could phosphorylates many pivotal downstream targets through the increase ratio of AMP/ATP in environment, affect metabolic pathways of cell growth, cell cycle and autophagy [33, 34]. In normal cells, the ratio of AMP/ATP is in balance condition, when it is disrupted, many diseases will be followed, as well as malignant disease [35–37]. The underlying genetic mechanisms are still not clearly that how *PRKAA1* gene affect the occurrence and development of cancer, one of them may be the activated AMPK and phosphorylated p53 induced cell cycle G1/S arrest, as well as influenced the cell cycle checkpoint [8]. On the other way, some publications reported that activated AMPK take pate in the activation of anti-inflammatory agents [38], as well as inhibition the inflammatory function of macrophage [39].The lack of anti-inflammatory function will occur with the allele mutation of *PRKAA1* allele, and the patients would suffer from several epithelium disease [40]. What’s more, another potential source of tumorigenesis is the bone marrow-derived cells, because they were reported could be recruited after epithelial damage [41]. Currently, several studies have demonstrated that *PRKAA1* polymorphisms conduce to the development of gastric cancer, Sun et al. [42] revealed that LINC00152/miR-139-5p could promote the cell glycolysis of gastric cancer cells through regulating *PRKAA1* expression, Li et al. [43] indicated that Calcium Binding Protein 39-Like (CAB39L) may be a novel potential regulator for gastric cancer metabolism, which function on activation of PRKAA1/2.

There are several studies concerned about the polymorphisms of *PRKAA1*, and the variant sites include rs154268, rs3805486, rs461404, rs6882903, rs13361707 and rs10074991. After our comprehensive search and analyses, only rs13361707 and rs10074991 have 3 or more studies, so the other studies are excluded. Among these publications, the result is not consistent. Li et al. [9] and Eom et al. [10] suggested that rs13361707 polymorphism is remarkable related to an upgraded risk to gastric cancer in Asian and Caucasian, respectively. However, Sun et al. [13] and Yuan et al. [15] indicated the controversial result, in their study, rs13361707 of *PRKAA1* doesn’t affect the process of gastric cancer. On the meanwhile, the first three study of Shi et al. indicated that T allele of rs13361707 shows a significantly increasing risk of GC, however, Li et al., Kim et al. and Cai et al. revealed that the mutant allele of rs13361707 (C allele) caused enhanced risk of GC. In another case-control study conducted by Dai et al. [16], they revealed that the polymorphism of rs13361707 also doesn’t work in esophageal squamous cell carcinoma. In current study, we identified that the rs13361707 polymorphism would not affect the susceptibility of cancer in the overall population. For rs10074991, Eom et al. [10] and Kim et al. [27] only reported partial increase cancer risks in several genetic models, while Campa et al. [12] revealed that rs10074991 is not associated with the tumorigenesis of breast cancer.

We revealed a significant decrease risk in allelic comparison model, heterozygote comparison model and dominant genetic model. Rs10074991 and rs13361707 polymorphisms both located at the intron of *PRKAA1*, within the perfect LD (R^2^ = 1.00). In a book named “Personalized Management of Gastric Cancer”, Zhu et al. [44] demonstrated that the rs13361707 LD block mainly spans PTGER4, TTC33, and *PRKAA1* gene, and a remarkable relationships between rs13361707 and these three genes were shown in the results from GTEx, so the polymorphism of rs13361707 might influence the expression of *PRKAA1*. In the current study, we enrolled all eligible case-control studies, from different race to different cancer types, aim to draw a systemic result for readers, and stratified analysis was also performed to avoid the bias. For example, the allele frequency of rs10074991, rs13361707 for all the enrolled Asian population based studies are ranged from 40 to 60%, while the Caucasian population based studies are ranged about 80% (Additional file 1: Table S1), it might cause by the ethnic difference. We exceed the Caucasian based study, and the overall result was not influenced (Table 2), but the future studies show focused on this difference between Asian and Caucasian. In the analysis of rs13361707, we also conducted the stratified analysis by the source of control, in order to reduce the heterogeneity of Q-test, however, the result in HB based subgroup or PB based subgroup is consistent with the result of overall pooled result. To confirm the result, large well-designed epidemiological studies based on population controls should be managed in the future.

There are several advantages of this meta-analysis. Initially, we enrolled all eligible studies focused on the relationships between PRKAA1 polymorphisms and overall cancer risks to conducted a comprehensive meta-analysis. Furthermore, NOS method was used to assess the quality of each accepted case-control study, and exceed the low quality studies to make sure the reliability of pooled result. Additionally, eligible studies were stratified and calculated in different subgroups to reduce the impact of heterogeneity, including subgroup of ethnicity, cancer type and source of control. Another point, the *P* value of Z was adjusted by Bonferroni corrections (*P*_*Adjust*_), aim to avoiding the false positive results. Finally, the stability of results was assessed by sensitivity analysis, and potential publication bias was eliminating from the results of Egger’s test and Begg’s funnel plot.

However, there are also several disadvantages. To begin with, the untrustworthy result may be obtained, because of lack of subjects in several genetic polymorphisms. What’s more, potential enrolled bias may be existed, due to that only publications written in English or Chinese were assessed. Then, most of the enrolled studies are concerned about GC, only 2 about ESCC, 1 about breast cancer, and 1 about lung cancer, therefore, the meta-analysis result might not be able to illustrated the impact of rs10074991 and rs13361707 in overall cancer risk. Last but not least, the striking level of heterogeneity between the enrolled publications might influence the result, although we conducted the meta-analysis with Der Simonian and Laird method.

## Conclusion

Our data have successfully elaborated that *PRKAA1* rs13361707 polymorphism is not participant with increased risk of cancer, while the A allele of *PRKAA1* rs10074991 revealed a significant decrease risk, especially in Asian population. In the future., larger sample size studies based on numerous cancer types should be conducted to confirm the exploration of this issue.

## Additional file

Additional file 1: **Table S1.** Total sample sizes and allelic percentages of enrolled studies. **Table S2.** Methodological quality of the included studies according to the Newcastle-Ottawa Scale. **Table S3.** PRISMA 2009 Checklist. **Table S4.** Details of the sensitivity analyses for the polymorphisms in *PRKAA1* and cancer risk. **Table S5.** *P* values of the Egger’s test for the polymorphisms in *PRKAA1*. **Figure S1.** Begg’s funnel plot for publication bias for *PRKAA1 rs13361707* polymorphism (B vs. A). For Begg’s funnel plot, the x-axis is log (OR), and the y-axis is natural logarithm of OR. The horizontal line in the figure represents the overall estimated log (OR). The two diagonal lines indicate the pseudo 95% confidence limits of the effect estimate. **Figure S2.** Begg’s funnel plot for publication bias for *PRKAA1 rs10074991* polymorphism (B vs. A). For Begg’s funnel plot, the x-axis is log (OR), and the y-axis is natural logarithm of OR. The horizontal line in the figure represents the overall estimated log (OR). The two diagonal lines indicate the pseudo 95% confidence limits of the effect estimate. **Figure S3.** Sensitivity analysis of Asian based group OR Co-efficient for PRKAA1 rs13361707 polymorphism (C vs. T). Results were calculated by omitting each study in turn. The two ends of the dotted lines represent the 95% CI. (PDF 1760 kb)

## Abbreviations

AMP: Adenosine monophosphate
AMPK: AMP-activated protein kinase
ATP: Adenosine triphosphate
CIs: Confidence intervals
HWE: Hardy-Weinberg Equilibrium
mTORC1: Mammalian target of rapamycin complex 1
ORs: Odds ratio
SNP: Single nucleotide polymorphism
TPM: Transcripts Per Kilobase Million
