# Supplementary material for: Do polymorphisms in protein kinase catalytic subunit alpha-1 gene associated with cancer susceptibility? a meta-analysis and systematic review
Source: BMC Med Genet. 2018 Oct 19;19:189. doi: 10.1186/s12881-018-0704-8 (PMC6194619; doi:10.1186/s12881-018-0704-8)
Supplement: Supplementary file 1 — Table S1. Total sample sizes and allelic percentages of enrolled studies. Table S2. Methodological quality of the included studies according to the Newcastle-Ottawa Scale. Table S3. PRISMA 2009 Checklist. Table S4. Details of the sensitivity analyses for the polymorphisms in PRKAA1 and cancer risk. Table S5. P values of the Egger’s test for the polymorphisms in PRKAA1. Figure S1. Begg’s funnel plot for publication bias for PRKAA1 rs13361707 polymorphism (B vs. A). For Begg’s funnel plot, the x-axis is log (OR), and the y-axis is natural logarithm of OR. The horizontal line in the figure represents the overall estimated log (OR). The two diagonal lines indicate the pseudo 95% confidence limits of the effect estimate. Figure S2. Begg’s funnel plot for publication bias for PRKAA1 rs10074991 polymorphism (B vs. A). For Begg’s funnel plot, the x-axis is log (OR), and the y-axis is natural logarithm of OR. The horizontal line in the figure represents the overall estimated log (OR). The two diagonal lines indicate the pseudo 95% confidence limits of the effect estimate. Figure S3. Sensitivity analysis of Asian based group OR Co-efficient for PRKAA1 rs13361707 polymorphism (C vs. T). Results were calculated by omitting each study in turn. The two ends of the dotted lines represent the 95% CI. (PDF 1760 kb) [file 12881_2018_704_MOESM1_ESM.pdf]

Supplementary Information

Do Polymorphisms in Protein Kinase Catalytic Subunit Alpha-1 Gene Associate with Cancer Susceptibility? A Meta-analysis and Systematic Review

Jialin Meng[1]; Xinyao Fan[2]; Meng Zhang[1]; Zongyao Hao[1]; Chaozhao Liang[1*]

[1]Department of Urology, The First Affiliated Hospital of Anhui Medical University; Institute of Urology, Anhui Medical University; Anhui Province Key Laboratory of Genitourinary Diseases, Anhui Medical University, Hefei, Anhui, China.

[2]Graduate School of Anhui Medical University, Hefei, Anhui, China.

Corresponding author: Chaozhao Liang, MD, PhD; Department of Urology, The First Affiliated Hospital of Anhui Medical University; Institute of Urology, Anhui Medical University; Anhui Province Key Laboratory of Genitourinary Diseases, Anhui Medical University, No.218, Jixi Road, Hefei 230022, Anhui, China;

Tel.: +86 55162923440;

Email address: liang_chaozhao@ahmu.edu.cn

Running Title: Association between *PRKAA1* variants and cancer susceptibility

Supplementary Table 1. Total sample sizes and allelic percentages of enrolled studies.

SNP	First author	Year	Cancer Type	Case			Control		
				n	Wild type allele	Mutant allele (%)	n	Wild type allele (%)	Mutant allele (%)
rs10074991	Campa *et al.*	2011	BC	1193	73.47%	26.53%	1873	71.65%	28.35%
rs10074991	Kim *et al.*	2014	GC	475	54.11%	45.89%	474	45.57%	54.43%
rs10074991	Eom *et al.*	2016	GC	846	54.20%	45.80%	846	45.33%	54.67%
rs13361707	Shi *et al.*	2011	GC	979	42.75%	57.25%	2268	52.20%	47.80%
rs13361707	Shi *et al.*	2011	GC	1873	44.93%	55.07%	2076	52.75%	47.25%
rs13361707	Shi *et al.*	2011	GC	1392	41.27%	58.73%	1513	50.53%	49.47%
rs13361707	Shi *et al.*	2011	GC	895	49.89%	50.11%	3227	47.13%	52.87%
rs13361707	Shi *et al.*	2011	GC	2404	55.51%	44.49%	3227	47.13%	52.87%
rs13361707	Li *et al.*	2013	GC	335	53.88%	46.12%	334	44.76%	55.24%
rs13361707	Song *et al.*	2013	GC	3245	53.50%	46.50%	1700	47.06%	52.94%
rs13361707	Dai *et al.*	2014	ESCC	2072	47.64%	52.36%	2254	47.87%	52.13%
rs13361707	Wu *et al.*	2014	GC	217	51.38%	48.62%	428	44.51%	55.49%
rs13361707	Kim *et al.*	2014	GC	475	54.21%	45.79%	473	45.88%	54.12%
rs13361707	Sun *et al.*	2014	GC	130	78.08%	21.92%	124	74.19%	25.81%
rs13361707	Dong *et al.*	2015	GC	167	42.51%	57.49%	186	53.49%	46.51%
rs13361707	Dong *et al.*	2015	NSCLC	158	48.42%	51.58%	186	53.49%	46.51%
rs13361707	Dong *et al.*	2015	ESCC	107	54.67%	45.33%	186	53.49%	46.51%
rs13361707	Qiu *et al.*	2015	GC	1124	56.01%	43.99%	1194	46.52%	53.48%
rs13361707	Zhang *et al.*	2016	GC	60	60.83%	39.17%	60	45.00%	55.00%
rs13361707	Eom *et al.*	2016	GC	846	54.31%	45.69%	846	45.63%	54.37%
rs13361707	Yuan *et al.*	2016	GC	116	52.16%	47.84%	102	51.47%	48.53%
rs13361707	Cai *et al.*	2017	GC	473	58.88%	41.12%	487	45.38%	54.62%

GC: Gastric cancer; BC: Breast cancer; ESCC: Esophageal squamous cell carcinoma; NSCLC: Non-small cell lung cancer;

Supplementary Table 2. Methodological quality of the included studies according to the Newcastle-Ottawa Scale.

Variants	Author	Year	Adequacy of Case Definition	Representativeness of the Caces	Selection of Controls	Definition of Controls	Comparability Cases/Controls	Ascertainment of Exposure	Same Method of Ascertainment	Non-response rate
rs10074991	Campa *et al.*	2011	*	*	*	*	**	*	*	NA
	Kim *et al.*	2014	*	*		*	*	*	*	NA
	Eom *et al.*	2016	*	*		*	**	*	*	NA
rs13361707	Shi *et al.*	2011	*	*	*	*	**	*	*	NA
	Shi *et al.*	2011	*	*	*	*	**	*	*	NA
	Shi *et al.*	2011	*	*	*	*	**	*	*	NA
	Shi *et al.*	2011	*	*	*	*	**	*	*	NA
	Shi *et al.*	2011	*	*	*	*	**	*	*	NA
	Li *et al.*	2013	*	*		*	**	*	*	NA
	Song *et al.*	2013	*	*	*	*	**	*	*	NA
	Dai *et al.*	2014	*	*		*	**	*	*	NA
	Wu *et al.*	2014	*	*		*	*	*	*	NA
	Kim *et al.*	2014	*	*		*	*	*	*	NA
	Sun *et al.*	2014	*	*	*	*	**	*	*	NA
	Dong *et al.*	2015	*	*		*	**	*	*	NA
	Dong *et al.*	2015	*	*		*	**	*	*	NA
	Dong *et al.*	2015	*	*		*	**	*	*	NA
	Qiu *et al.*	2015	*	*	*	*	**	*	*	NA
	Zhang *et al.*	2016	*	*	*	*	**	*	*	NA
	Eom *et al.*	2016	*	*		*	**	*	*	NA
	Yuan *et al.*	2016	*	*	*	*	**	*	*	NA
	Cai *et al.*	2017	*	*	*	*	*	*	*	NA

Supplementary table 3 :PRISMA 2009 Checklist

Section/topic	#	Checklist item	Reported on page #
**TITLE**			
Title	1	Identify the report as a systematic review, meta-analysis, or both.	Page 1
**ABSTRACT**			
Structured summary	2	Provide a structured summary including, as applicable: background; objectives; data sources; study eligibility criteria, participants, and interventions; study appraisal and synthesis methods; results; limitations; conclusions and implications of key findings; systematic review registration number.	Page 2
**INTRODUCTION**			
Rationale	3	Describe the rationale for the review in the context of what is already known.	Page 3
Objectives	4	Provide an explicit statement of questions being addressed with reference to participants, interventions, comparisons, outcomes, and study design (PICOS).	Page 3
**METHODS**			
Protocol and registration	5	Indicate if a review protocol exists, if and where it can be accessed (e.g., Web address), and, if available, provide registration information including registration number.	N/A
Eligibility criteria	6	Specify study characteristics (e.g., PICOS, length of follow-up) and report characteristics (e.g., years considered, language, publication status) used as criteria for eligibility, giving rationale.	Study selection: Page 4
Information sources	7	Describe all information sources (e.g., databases with dates of coverage, contact with study authors to identify additional studies) in the search and date last searched.	Search strategy: Page 4
Search	8	Present full electronic search strategy for at least one database, including any limits used, such that it could be repeated.	Search strategy: Page 4
Study selection	9	State the process for selecting studies (i.e., screening, eligibility, included in systematic review, and, if applicable, included in the meta-analysis).	Figure 1
Data collection process	10	Describe method of data extraction from reports (e.g., piloted forms, independently, in duplicate) and any processes for obtaining and confirming data from investigators.	Data extraction: Page 4
Data items	11	List and define all variables for which data were sought (e.g., PICOS, funding sources) and any assumptions and simplifications made.	Data extraction: Page 4
Risk of bias in individual studies	12	Describe methods used for assessing risk of bias of individual studies (including specification of whether this was done at the study or outcome level), and how this information is to be used in any data synthesis.	Statistical analysis: Page5-6
Summary measures	13	State the principal summary measures (e.g., risk ratio, difference in means).	Statistical analysis: Page5-6
Synthesis of results	14	Describe the methods of handling data and combining results of studies, if done, including measures of consistency (e.g., I^2) for each meta-analysis.	Statistical analysis: Page5-6

Supplementary table 3 :PRISMA 2009 Checklist

Section/topic	#	Checklist item	Reported on page #
Risk of bias across studies	15	Specify any assessment of risk of bias that may affect the cumulative evidence (e.g., publication bias, selective reporting within studies).	Statistical analysis: Page5-6
Additional analyses	16	Describe methods of additional analyses (e.g., sensitivity or subgroup analyses, meta-regression), if done, indicating which were pre-specified.	Statistical analysis: Page5-6
RESULTS			
Study selection	17	Give numbers of studies screened, assessed for eligibility, and included in the review, with reasons for exclusions at each stage, ideally with a flow diagram.	Description of studies: page 6-7
Study characteristics	18	For each study, present characteristics for which data were extracted (e.g., study size, PICOS, follow-up period) and provide the citations.	Table 1-2
Risk of bias within studies	19	Present data on risk of bias of each study and, if available, any outcome level assessment (see item 12).	Page 6-7
Results of individual studies	20	For all outcomes considered (benefits or harms), present, for each study: (a) simple summary data for each intervention group (b) effect estimates and confidence intervals, ideally with a forest plot.	Page 6-7
Synthesis of results	21	Present results of each meta-analysis done, including confidence intervals and measures of consistency.	Page 6-7
Risk of bias across studies	22	Present results of any assessment of risk of bias across studies (see Item 15).	Page 6-7
Additional analysis	23	Give results of additional analyses, if done (e.g., sensitivity or subgroup analyses, meta-regression [see Item 16]).	Page 7
DISCUSSION			
Summary of evidence	24	Summarize the main findings including the strength of evidence for each main outcome; consider their relevance to key groups (e.g., healthcare providers, users, and policy makers).	Page 8
Limitations	25	Discuss limitations at study and outcome level (e.g., risk of bias), and at review-level (e.g., incomplete retrieval of identified research, reporting bias).	Page 9
Conclusions	26	Provide a general interpretation of the results in the context of other evidence, and implications for future research.	Page 9
FUNDING			
Funding	27	Describe sources of funding for the systematic review and other support (e.g., supply of data); role of funders for the systematic review.	Page 10

From: Moher D, Liberati A, Tetzlaff J, Altman DG, The PRISMA Group (2009). Preferred Reporting Items for Systematic Reviews and Meta-Analyses: The PRISMA Statement. PLoS Med 6(7): e1000097. doi:10.1371/journal.pmed1000097

For more information, visit: **www.prisma-statement.org**.

Supplementary Table 4. Details of the sensitivity analyses for the polymorphisms in *PRKAA1* and cancer risk.

SNP	Comparison	Study omitted	Estimate	[95% Confident Interval]	Effect Model
rs10074991	B VS. A	Campa *et al.(2011)*	0.704	0.632-0.785	Random
		Kim *et al.*l.*(2014)*	0.802	0.619-1.038	
		Eom *et al.(2016)*	0.813	0.636-1.039	
		Combined	0.773	0.642-0.931	
	BB vs. AA	Campa *et al.(2011)*	0.489	0.392-0.609	Random
		Kim *et al.*l.*(2014)*	0.671	0.357-1.261	
		Eom *et al.(2016)*	0.684	0.369-1.268	
		Combined	0.610	0.393-0.946	
	BA vs. AA	Campa *et al.(2011)*	0.675	0.558-0.816	Fixed
		Kim *et al.*l.*(2014)*	0.795	0.699-0.904	
		Eom *et al.(2016)*	0.820	0.714-0.941	
		Combined	0.779	0.691-0.877	
	BB+BA vs. AA	Campa *et al.(2011)*	0.607	0.507-0.727	Random
		Kim *et al.*l.*(2014)*	0.730	0.511-1.042	
		Eom *et al.(2016)*	0.751	0.54-1.044	
		Combined	0.697	0.553-0.912	
	BB vs. BA+AA	Campa *et al.(2011)*	0.638	0.534-0.762	Random
		Kim *et al.*l.*(2014)*	0.789	0.513-1.213	
		Eom *et al.(2016)*	0.798	0.519-1.228	
		Combined	0.780	0.517-1.177	
rs13361707	B VS. A	Shi *et al. (2011)*	0.873	0.756-1.007	Random
		Shi *et al.(2011)*	0.876	0.758-1.012	
		Shi *et al.(2011)*	0.873	0.757-1.007	
		Shi *et al.(2011)*	0.899	0.767-1.053	
		Shi *et al.(2011)*	0.912	0.783-1.063	
		Li *et al.(2013)*	0.912	0.784-1.062	
		Song *et al.(2013)*	0.907	0.775-1.062	
		Dai *et al.(2013)*	0.892	0.759-1.048	
		Wu *et al.(2014)*	0.907	0.779-1.057	
		Kim *et al.(2014)*	0.911	0.782-1.061	
		Sun *et al.(2014)*	0.903	0.777-1.050	
		Dong *et al.(2015)*	0.875	0.753-1.016	
		Dong *et al.(2015)*	0.885	0.761-1.030	
		Dong *et al.(2015)*	0.896	0.770-1.043	
		Qiu *et al.(2015)*	0.914	0.786-1.064	
		Zhang *et al.(2016)*	0.916	0.789-1.064	
		Eom *et al.(2016)*	0.912	0.783-1.063	
		Yuan *et al.(2016)*	0.896	0.770-1.042	
		Cai *et al.(2017)*	0.922	0.795-1.071	
		Combined	0.899	0.776-1.042	
	BB vs. AA	Shi *et al.(2011)*	0.761	0.570-1.015	Random
		Shi *et al.(2011)*	0.769	0.574-1.029	
		Shi *et al.(2011)*	0.763	0.571-1.021	
		Shi *et al.(2011)*	0.809	0.587-1.116	

	Shi *et al.(2011)*	0.834	0.613-1.136	
	Li *et al.(2013)*	0.834	0.613-1.134	
	Song *et al.(2013)*	0.825	0.599-1.135	
	Dai *et al.(2013)*	0.797	0.575-1.104	
	Wu *et al.(2014)*	0.825	0.606-1.123	
	Kim *et al.(2014)*	0.832	0.611-1.133	
	Sun *et al.(2014)*	0.816	0.602-1.106	
	Dong *et al.(2015)*	0.769	0.567-1.042	
	Dong *et al.(2015)*	0.785	0.578-1.067	
	Dong *et al.(2015)*	0.805	0.592-1.094	
	Qiu *et al.(2015)*	0.838	0.616-1.139	
	Zhang *et al.(2016)*	0.842	0.621-1.138	
	Eom *et al.(2016)*	0.834	0.612-1.136	
	Yuan *et al.(2016)*	0.804	0.592-1.092	
	Cai *et al.(2017)*	0.851	0.629-1.151	
	Combined	0.810	0.601-1.091	
BA vs. AA	Shi *et al.(2011)*	0.865	0.746-1.003	Random
	Shi *et al.(2011)*	0.873	0.747-1.019	
	Shi *et al.(2011)*	0.870	0.745-1.017	
	Shi *et al.(2011)*	0.899	0.758-1.066	
	Shi *et al.(2011)*	0.911	0.771-1.077	
	Li *et al.(2013)*	0.912	0.774-1.073	
	Song *et al.(2013)*	0.904	0.762-1.074	
	Dai *et al.(2013)*	0.890	0.749-1.058	
	Wu *et al.(2014)*	0.900	0.764-1.061	
	Kim *et al.(2014)*	0.913	0.775-1.075	
	Sun *et al.(2014)*	0.903	0.768-1.063	
	Dong *et al.(2015)*	0.892	0.758-1.050	
	Dong *et al.(2015)*	0.894	0.760-1.053	
	Dong *et al.(2015)*	0.899	0.764-1.057	
	Qiu *et al.(2015)*	0.906	0.765-1.071	
	Zhang *et al.(2016)*	0.919	0.784-1.076	
	Eom *et al.(2016)*	0.915	0.776-1.078	
	Yuan *et al.(2016)*	0.894	0.760-1.051	
	Cai *et al.(2017)*	0.934	0.799-1.091	
	Combined	0.900	0.768-1.054	
BB+BA vs. AA	Shi *et al.(2011)*	0.829	0.685-1.002	Random
	Shi *et al.(2011)*	0.837	0.689-1.017	
	Shi *et al.(2011)*	0.833	0.686-1.012	
	Shi *et al.(2011)*	0.868	0.700-1.077	
	Shi *et al.(2011)*	0.883	0.716-1.090	
	Li *et al.(2013)*	0.885	0.720-1.087	
	Song *et al.(2013)*	0.877	0.706-1.088	
	Dai *et al.(2013)*	0.858	0.690-1.068	
	Wu *et al.(2014)*	0.874	0.711-1.076	
	Kim *et al.(2014)*	0.884	0.719-1.088	
	Sun *et al.(2014)*	0.872	0.710-1.072	
	Dong *et al.(2015)*	0.847	0.690-1.040	

	Dong *et al.(2015)*	0.856	0.696-1.052	
	Dong *et al.(2015)*	0.866	0.705-1.064	
	Qiu *et al.(2015)*	0.882	0.715-1.088	
	Zhang *et al.(2016)*	0.894	0.731-1.094	
	Eom *et al.(2016)*	0.886	0.720-1.090	
	Yuan *et al.(2016)*	0.862	0.702-1.058	
	Cai *et al.(2017)*	0.904	0.740-1.104	
	Combined	0.868	0.711-1.061	
BB vs. BA+AA	Shi *et al.(2011)*	0.841	0.688-1.029	Random
	Shi *et al.(2011)*	0.843	0.690-1.031	
	Shi *et al.(2011)*	0.840	0.688-1.024	
	Shi *et al.(2011)*	0.873	0.702-1.087	
	Shi *et al.(2011)*	0.895	0.727-1.102	
	Li *et al.(2013)*	0.891	0.723-1.099	
	Song *et al.(2013)*	0.888	0.715-1.102	
	Dai *et al.(2013)*	0.865	0.692-1.080	
	Wu *et al.(2014)*	0.889	0.721-1.097	
	Kim *et al.(2014)*	0.890	0.721-1.098	
	Sun *et al.(2014)*	0.878	0.715-1.079	
	Dong *et al.(2015)*	0.836	0.681-1.027	
	Dong *et al.(2015)*	0.852	0.692-1.049	
	Dong *et al.(2015)*	0.870	0.706-1.071	
	Qiu *et al.(2015)*	0.902	0.734-1.107	
	Zhang *et al.(2016)*	0.886	0.721-1.088	
	Eom *et al.(2016)*	0.891	0.722-1.101	
	Yuan *et al.(2016)*	0.873	0.709-1.075	
	Cai *et al.(2017)*	0.898	0.729-1.106	
	Combined	0.874	0.713-1.070	

Supplementary Table 5. *P* values of the Egger's test for the polymorphisms in *PRKAA1*.

Polymorphisms	Subgroup	Egger's test P > |t|
rs10074991	overall	0.481
rs13361707	overall	0.710
	Asian	0.738
	GC	0.657
	PB	0.846
	HB	0.053

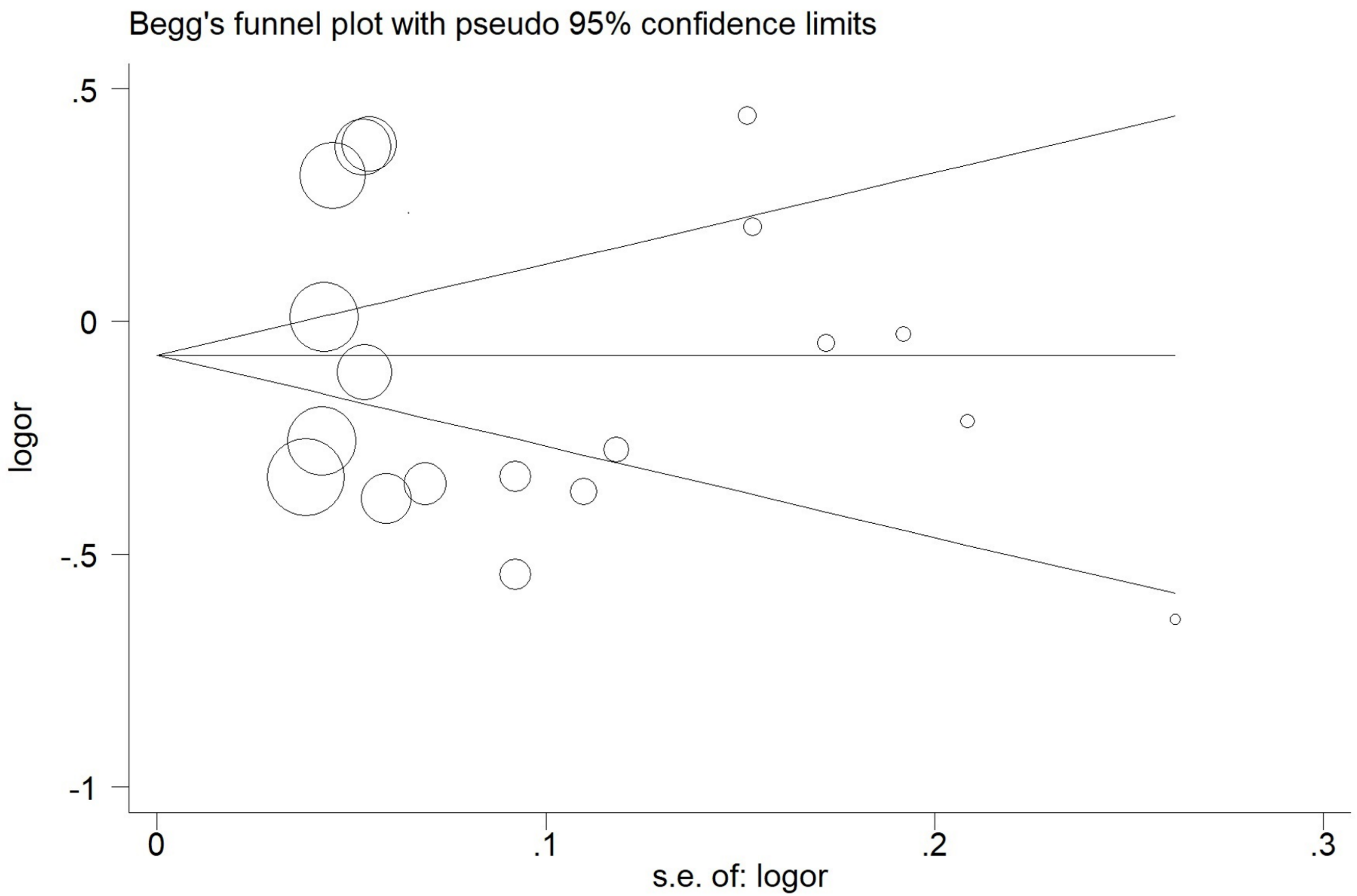

Supplementary Figure 1. Begg's funnel plot for publication bias for PRKAA1 rs13361707 polymorphism (B vs. A). For Begg's funnel plot, the x-axis is log (OR), and the y-axis is natural logarithm of OR. The horizontal line in the figure represents the overall estimated log (OR). The two diagonal lines indicate the pseudo 95% confidence limits of the effect estimate.

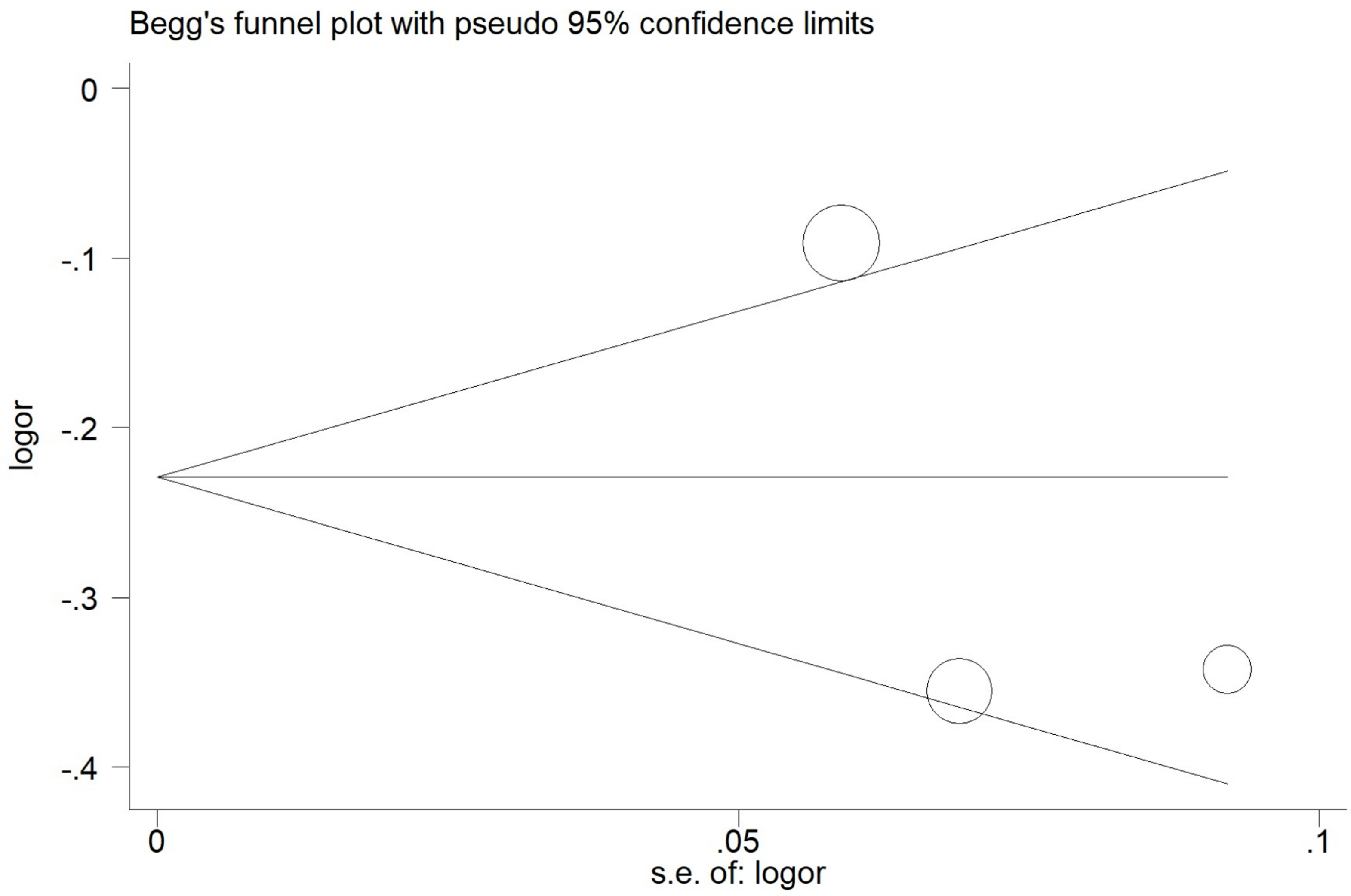

Supplementary Figure 2. Begg's funnel plot for publication bias for PRKAA1 rs10074991 polymorphism (B vs. A). For Begg's funnel plot, the x-axis is log (OR), and the y-axis is natural logarithm of OR. The horizontal line in the figure represents the overall estimated log (OR). The two diagonal lines indicate the pseudo 95% confidence limits of the effect estimate.

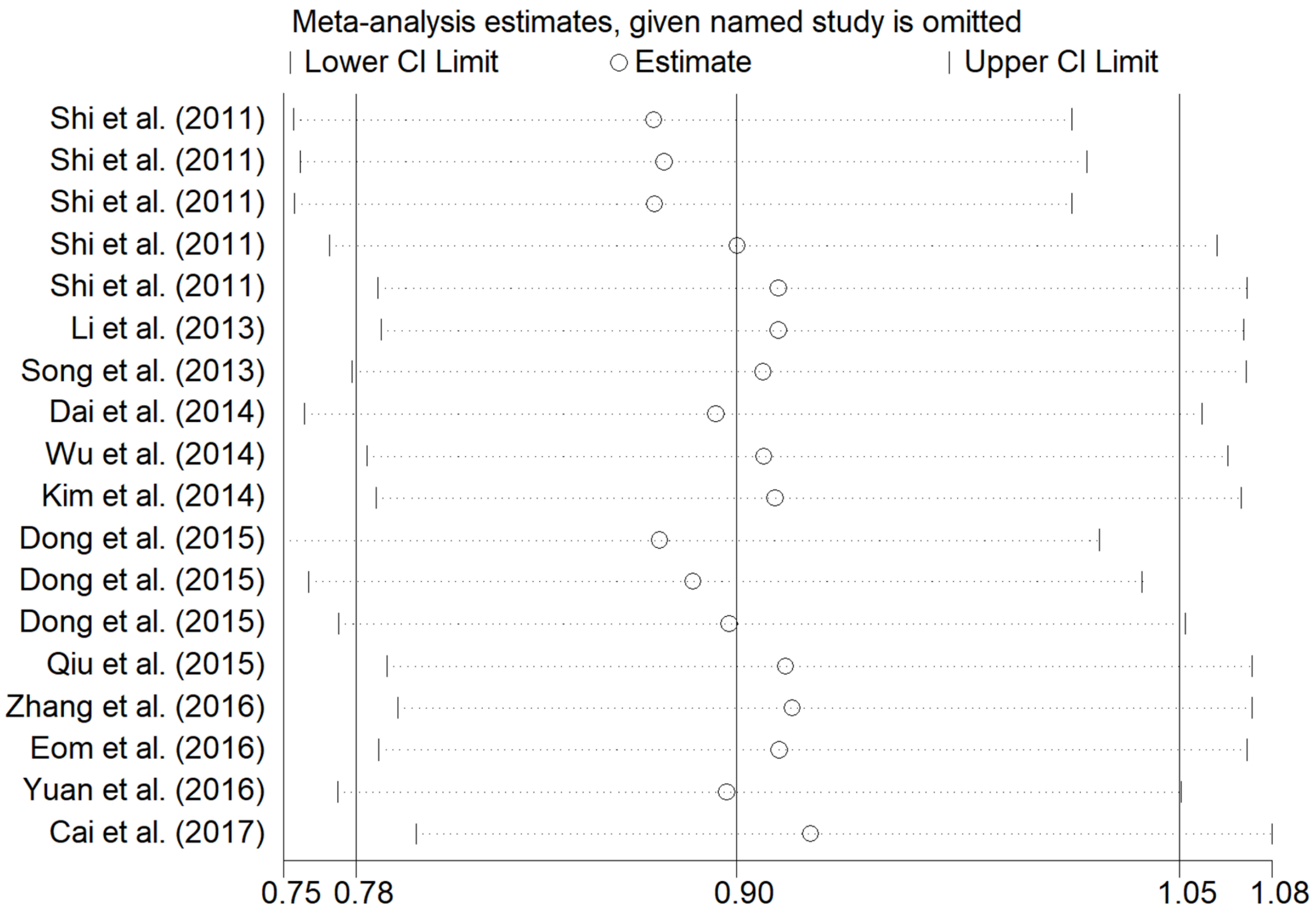

Supplementary Figure 3. Sensitivity analysis of Asian based group OR Co-efficient for PRKAA1 rs13361707 polymorphism (C vs. T). Results were calculated by omitting each study in turn. The two ends of the dotted lines represent the 95%CI.
